# Predictors of Pain Recurrence After Lumbar Facet Joint Injections

**DOI:** 10.3389/fnins.2019.00958

**Published:** 2019-09-20

**Authors:** Wuilker Knoner Campos, Marcelo Neves Linhares, Jamir Sarda, Adair Roberto Soares Santos, Kátia Lin, Alexandra Latini, Roger Walz

**Affiliations:** ^1^Functional Neurosurgery Division, Department of Neurosurgery, Baia Sul Medical Center, Florianópolis, Brazil; ^2^Neuron Institute, Baia Sul Medical Center, Florianópolis, Brazil; ^3^Neurosurgery Division, Hospital Governador Celso Ramos, Florianópolis, Brazil; ^4^Center for Applied Neuroscience (CeNAp), University Hospital (HU), Federal University of Santa Catarina (UFSC), Florianópolis, Brazil; ^5^Department of Surgery, Neurosurgery Division, HU, UFSC, Florianópolis, Brazil; ^6^Department of Psychology and Master Program of Health and Work, Univali, Itajaí, Brazil; ^7^Department of Physiology, UFSC, Florianópolis, Brazil; ^8^Department of Internal Medicine, Neurology Division, HU, UFSC, Florianópolis, Brazil; ^9^Laboratory of Bioenergetics and Oxidative Stress, Department of Biochemistry, Federal University of Santa Catarina (UFSC), Florianópolis, Brazil

**Keywords:** spine, facet joint injection, low back pain, facet joint pain, disability, catastrophizing, psychological factors

## Abstract

**Introduction:**

Facet joint injections (FJIs) of anesthetic and corticosteroids are useful for the diagnosis and treatment of low back pain (LBP). In the current study, we evaluated the efficacy of FJI on LBP treatment and the predictive variables of pain recurrence after FJI.

**Methods:**

We included and followed prospectively forty-three consecutive patients with chronic LBP treated with FJI. Clinical assessments were carried out at a baseline 1 week before FJIs and after a 6-month follow-up visit using the visual analog scale (VAS) for pain, Oswestry Disability Index (ODI) for disability-specific measure and MacNab criteria for global effectiveness, and compared through analysis using paired-samples “*t*” tests. Multiple cox-regression analysis was used to identify the presurgical variables independently associated with pain recurrence anytime during the follow-up. In addition to the demographic, clinical, and surgical data, we also analyzed psychometric scales: Pain Catastrophizing Scale (PCS), Beck Anxiety Inventory (BAI), and Beck Depression Inventory (BDI).

**Results:**

After a 6-month follow-up, thirty-two patients (74.4%) showed a clinically significant reduction of pain and twenty-seven (62.8%) reported a clinically significant improvement of disability. Presurgical catastrophizing (PCS score ≥ 5, adjusted HR 4.4, CI 95% 1.7–11.3, *p* = 0.002) and smoking (Adjusted HR 12.5, CI 95% 1.1–138.9, *p* = 0.04) remains associated with pain recurrence.

**Conclusion:**

FJI reduces LBP and disability of patients with unresponsive LBP. Pain-related cognitive and behavioral factors determined by pain catastrophizing and smoking were independently associated with pain recurrence after lumbar FJI. The results support the need of a multidisciplinary approach for presurgical evaluation of patients with chronic pain.

## Introduction

According to authors, low back pain (LBP) is responsible for more years lived with a disability than any other disorder (Vos et al., 2012). Several anatomical parts in the lower back are associated with LBP, like muscles, fascia, intervertebral discs, ligaments, and facet and sacroiliac joints (Manchikanti et al., 2001, 2015). According to biomechanical studies and controlled studies with facet joint injection (FJI), facet joints are considered the most usual location of pain, with about 40% of the LBP cases (Datta et al., 2009; Falco et al., 2012).

Anatomically, facet joints are structured in superior articular processes paired with inferior articular processes of adjacent vertebrae that allow spine flexion and rotation. The nerve endings from medial branches of the dorsal rami innervate the facet joints (Cavanaugh et al., 2006; Bogduk, 2010; Manchikanti et al., 2013). Authors have been proposed that degeneration of the facet joint may result from combined asymmetrical motion with spondylolisthesis, analogous the other synovial joints (Eubanks et al., 2007; Kalichman et al., 2008; Maataoui et al., 2014). Therefore, the rationale for the basis of facet joint pain is the presence of an osteophyte impacting on a nerve, a stretching of the joint capsule, constricting of synovial villi of articular surfaces, and inflammatory chemicals released in facet joint (Igarashi et al., 2004; Gellhorn et al., 2013; Kras et al., 2014).

For the management of lumbar facet joint pain, in particular pain that is unresponsive to conservative therapy, FJI has been suggested (Manchikanti et al., 2010) for diagnostic examination (symptomatic/asymptomatic facet joint differentiation) and for pain management in patients with painful facet joint syndrome (Kelekis et al., 2005; Falco et al., 2012; Filippiadis et al., 2014; Santiago et al., 2014). The rationale for the indication of FJI is that the injection of corticosteroids has been shown to be effective in the short term (4 weeks) at producing benefits for a range of musculoskeletal disorders in other joints (Zhang et al., 2005; Bellamy et al., 2006). The pain relief may facilitate exercises designed to improve muscular strength and range of movement on lumbar spine (Chou et al., 2011). FJI may be useful in symptomatic spondylolysis (Kang et al., 2018).

It is well-known that persistent LBP is associated with biopsychosocial factors. In patients with pain continuing for more than 6 months, despite optimized conservative treatment and FJI, chronic pain and physical disability are common symptoms. For this motive, a therapy as the FJIs may not resolve this pain disorder completely. Recently, there has been an increase in findings on the psychosocial aspects (e.g., depression, catastrophizing, pain sensitivity) as predictors for surgical outcomes among patient with chronic pain (Flor and Turk, 1989; Khan et al., 2011; Campos et al., 2019).

One retrospective study (follow-up surveys returned by 86 of 166 patients, 52%) shows that in medial branch blocks (MBBs) for chronic low back (90% of cases) or neck pain, the level of anxiety and depressive symptoms accessed by the Hospital Anxiety and Depression Scale (HADS) was negatively and independently associated with lower probability to reach a clinically meaningful difference in pain 1 month after the procedure (Wasan et al., 2009). To our knowledge, there is no prospective study investigating the predictive value of psychological symptoms for clinically meaningful pain and quality of life improvement and pain recurrence after the LBP treatment with FJI.

This study aimed to examine: (i) the incidence of meaningful clinical improvement of pain and disability 6 months after FJI for chronic LBP unresponsive to conservative therapy; (ii) the predictive value of psychological symptoms and pain-related cognitions for pain recurrence after the procedure. We hypothesized that the psychological symptoms (depression, anxiety) and pain-related cognitions independently associated with a higher incidence of pain recurrence after the FJI to treat LBP.

## Materials and Methods

### Patient Selection

Patients over 18 years of age with chronic LBP for at more than 3 months in duration and consecutively referred to our pain clinic between January 2012 and January 2014. Facet joint pain was considered to be present by the neurosurgeon evaluation when: (i) no radicular symptoms (described as pain spreading below the knee); (ii) no sacroiliac joint pain (after a pain provocation test); (iii) lumbar paraspinal palpation with increased pain; and (iv) increased pain on one or more of the following: (a) extension (more than flexion)/rotation, (b) extension/side flexion, and (c) extension/rotation (Sandhu et al., 2015). Patients had failed conservative therapy before starting interventional pain procedures. Exclusion criteria were any malignancy, infection, or inflammatory spine disorders. The pain team periodically reviewed the LBP diagnosis according to the 2009 NICE guidance for the management of LBP (Savigny et al., 2009). The multidisciplinary team was comprised of a pain consultant, neurosurgeon, psychologist, and physiotherapist to evaluate the different pain domains. FJI procedure was a part of standard care recommended by the neurosurgeon of the multidisciplinary team.

### Surgical Procedure

Under sedation and with the patient in the prone position, local anesthesia (1% lidocaine) was delivered using a 25-gauge hypodermic needle at the entry point according to landmarks of fluoroscopic guidance (scotty-dog view). The injection syringe containing 10 ml of Ropivacaine (10 mg/ml) and 2 ml of Diprospan^®^ suspension equivalent to 7 mg/mL of betamethasone (dipropionato 6.43 mg/mL and disodium phosphate 2.63 mg/mL) was prepared by the surgeon.

All patients had bilateral facet joint pain at levels L4/L5 and L5/S1, which could be symmetric (both sides equally painful) or asymmetric (pain predominance in one side). In all cases, facet joints of L3/L4, L4/L5, and L5/S1 levels were injected bilaterally with 2 ml through the 22-gauge spinal needle placed into each joint. After injections, the spinal needle was removed, and a sterile bandage was applied.

### Presurgical Demographic, Clinical Variables, and Follow-Up

The presurgical demographic, clinical and radiological variables were recorded by the pain consultant and the neurosurgeon trough a research protocol and included: age, sex, body mass index (BMI), years of education, occupational activity, smoke habits, diabetes mellitus, fibromyalgia, hypertension, sedentary lifestyle, previous opioid use, previous lumbar surgery, pain duration, pain locality, and magnetic resonance image (MRI) findings. The pain consultant made the diagnosis of fibromyalgia, and the diagnosis of hypertension and type II diabetes were confirmed by contact with the primary care physician. Patients were considered with sedentary lifestyle if they do less than 450 metabolic equivalents per week, as supported by the American College of Sports Medicine and the American Heart Association (Haskell et al., 2007).

Pre-operatively levels of psychological symptoms were determined by a nurse under psychologist supervision, both with long-term experience in the field of chronic pain. Pain catastrophizing symptoms were evaluated using the Brazilian version of the Pain Catastrophizing Scale (PCS) (Caumo et al., 2002), which consists of nine items scored as a Likert scale, which varies from 0 to 5 points associated to the words “almost never” and “almost always” at the extremities. The total score is the sum of all items divided by the number of answered items, and the minimum score can be zero (0) and the maximum 5. Higher scores indicate the presence of catastrophizing thoughts. The anxiety and depression symptoms were evaluated using the Brazilian Version of Beck Anxiety Inventory (BAI) and Beck Depression Inventory (BDI), respectively (Beck et al., 1988; Gorenstein et al., 1999; Andrade et al., 2001; Caumo et al., 2002). The BAI and BDI consists of 21 items in each inventory, including symptoms and attitudes whose intensity ranges from neutral to a maximum level of severity, rated as 0–3. Higher scores indicate more anxiety or depressive symptoms, respectively (Table 1).

**TABLE 1:** Demographic, clinical and radiological characteristics of 43 patients with LBP treated with FJI.

**Continuous variables**	**Mean (SD)**
Age (years)	52 (16.8)
BMI (kg m^–2^)^a^	27.0 (2.5)
Duration of pain (months)	46.2 (48.0)
PCS^b^	8.9 (8.3)
BAI^c^	6.6 (6.5)
BDI^d^	7.2 (6.7)
Duration of pain relief (months)	7.6 (8.9)

**Categorical variables**	***n* (%)**

Sex	
Female	25 (58.1)
Male	18 (41.9)
Years of education	
<12 years	27 (62.8)
≥12 years	16 (37.2)
Type of occupational activity	
Non-manual work	30 (69.8)
Manual work	13 (30.2)
Obesity, BMI > 30 (kg m^–2^)^a^	07 (16.3)
Smoking	01 (2.3)
Diabetes mellitus II	03 (7.0)
Fibromyalgia	07 (16.3)
Hypertension	18 (41.9)
Sedentary life style	29 (67.4)
Opioid use before FJI	13 (30.2)
Previous lumbar surgery	09 (21.0)
Microdiscectomy	06 (14.0)
Decompression	01 (2.3)
Other	02 (4.7)
Arthrodesis surgery	06 (14.0)
**MR findings before FJI**	
Level of facet joint arthropathy	
L3–L4	01 (2.3)
L4–L5	05 (11.6)
L5–S1	04 (9.3)
L2–S1	07 (16.3)
L3–S1	20 (46.5)
L4–S1	06 (14.0)
Spine deformity	19 (44.2)
Flat back	12 (27.9)
Kyphosis	01 (2.3)
Scoliosis	04 (9.3)
Hyperlordosis	02 (4.7)
Degenerative disc disease	39 (90.7)
Black disc	32 (74.4)
Modic phenomenon	07 (16.3)
Disc herniation	30 (69.8)
Protrusion disc	26 (60.5)
Bulging disc	03 (7.0)
Extrusion disc	01 (2.3)
Spondylolisthesis	13 (30.2)
Facet synovial cyst	02 (4.7)
Lumbar muscle weakness	13 (30.2)
Supraspinal ligament stretch	15 (34.9)
Spinal stenosis	13 (30.2)
Oswestry Disability Index before the FJI	
Moderate functional disability (ODI 21–40%)	15 (35.0)
Severe functional disability (ODI 41–61%)	14 (32.5)
Crippled (ODI 61–81%)	14 (32.5)
**Surgical results**	
MacNab criteria, 6-months follow-up after FJI	
Excellent	09 (20.9)
Good	26 (60.5)
Fair	04 (9.3)
Poor	04 (9.3)
Complications	08 (18.6)
Bleeding	01 (2.3)
Lower limb block	06 (14.0)
Vesical retention	01 (2.3)

*^*a*^BMI = Body mass index; ^*b*^PCS = Pain Catastrophizing Scale; ^*c*^BAI = Beck Anxiety Inventory; ^*d*^Beck Depression Inventor.*

After FJI, patients received follow-up assessments by the neurosurgeon at 1, 3, and 6 months and afterward yearly. The primary outcomes were a clinically significant improvement of pain and disability assessed at 6 months, and pain recurrence up to 36 months follow-up after the procedure.

### Outcome Measures

Assessments were carried out at baseline 1 week before FJIs and after a 6-month follow-up visit. Patients were asked to rate their average pain intensity on a 100-mm visual analog scale (VAS) 31, with the endpoints “*no pain*” and “*worst possible pain* (Price et al., 1983).” Pain intensity was estimated using the VAS scale measured for three post-operative days and immediately at the time of pre-injection. The Oswestry Disability Index (ODI) determined the disability-specific measure (Fairbank and Pynsent, 2000; Fairbank et al., 2000). Patients also rated the global effectiveness of FJI using the MacNab criteria (poor, fair, good, excellent) (Macnab, 1971).

The neurosurgeon followed the patients for pain recurrence at the expected follow-up visits or any time when the patient required because of pain recurrence (additional visits). For the survival analysis proposes, patients showing no clinically meaningful improvement of pain on the first day after the procedure were classified as having a pain recurrence. Recurrence was also considered for those patients showing an initial clinically meaningful improvement of VAS but increase the VAS score again in five or more points during at least 24 h. This 5-point cutoff was determined based on the clinically significant change in the VAS score determined in our sample of patients (see below the results). The month of pain recurrence was used to determine the survival curve analysis (see below the statistical analysis). For patients without any improvement immediately and later after the procedure, the follow-up up duration (in months) without pain was considered zero.

### Statistical Analysis

Paired-samples “*t*” tests analyzed comparisons between the mean (SD) scores of VAS and ODI scales before and 6 months after FJI. We determined that the amount of change in the VAS and ODI scales by the difference between post-minus pre-surgical scores of VAS and the ODI scales, and the amount of change in VAS and ODI scores to be considered clinically significant were determined based on patient-centered estimation as previously described by our group (Campos et al., 2019). Therefore, we calculated the mean (SD) and the confidence interval of 95% (CI 95%) of the difference between the post-minus pre-surgical scores in the VAS and the ODI scales for each group of patients divided according to the MacNab category of global effectiveness. The inferior limit of the CI 95% of VAS and ODI scales calculated for the group of patients reporting MacNab criteria of good or excellent global effectiveness after the SCS was used to establish the amount of change in pain and disability necessary to be considered clinically significant (Campos et al., 2019).

We analyzed the time-course until the pain recurrence rose to a level considered unacceptable by the patient. For patients who did not report pain recurrence, we considered the total post-operative follow-up period in the analysis. We showed the cumulative probability of remaining pain-relieved by the Kaplan–Meyer survival curve. We used the univariate cox regression to identify presurgical demographics, clinical, neuroimaging, and level of emotional symptoms associated with pain recurrence after the FJI. All variables showing an association with pain recurrence with a “*p*” < 0.20 level of significance in the univariate analysis were included in the multiple Cox regressions to identify the independent predictors of pain recurrence after IFJ. We determined the level of association between the predictive variables and pain recurrence by the hazard ratio and the respective confidence interval of 95% (HR, CI 95%). Because all the observed associations showed clinical and biological plausibility and to avoid a type II error, we did not make corrections for multiple comparisons and a “*p*” level < 0.05 was considered significant. Statistical analysis was done using SPSS software (version 17.0, SPSS Inc., Chicago, IL, United States).

## Results

Forty-three patients underwent FJI. Table 1 shows the demographic and clinical variables. Twenty-five patients (58.1%) were female, the mean age was 52, and the mean duration of pain was 46 months. Regarding the pre-operative ODI scores, 15 patients (35%) had a moderate functional disability, 14 patients (32.5%) had a severe functional disability, and 14 patients (32.5%) were crippled. None of the patients became worse after the FJI.

**FIGURE 1:**
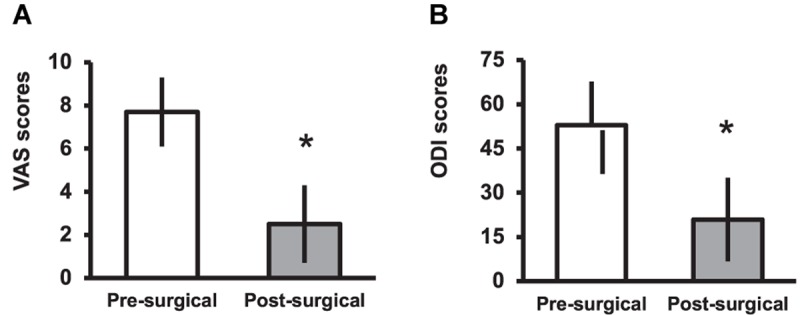
**(A)** Visual analog scale (VAS) and **(B)** Oswestry Disability Index (ODI) mean (SD) scores before and 6 months after facet joint injections for low back pain. ^∗^Statistical significant difference between post- and pre-surgical scores for a “*p*” < 0.0001 level determined by paired-samples “*t*” test.

Using the MacNab criteria, the global effectiveness of the FJI at the 6-month follow-up was “good” in 26 patients (60.5%); “excellent” in 9 (20.9%), “fair” in 4 (9.3%), and “poor” in 4 (9.3%) patients. There were 18 patients (41%) who became pain-free until the last follow-up visit, ranging from 6 to 36 months (12 ± 2, mean ± SE). There was a significant reduction of pain (*p* < 0.0001) evaluated by mean VAS scores (from 7.9 to 2.5, Figure 1A) and disability determined by mean ODI scores (reduction from 52.8 to 20.8, Figure 1B). The amount of pain reduction and improvement of disability necessary to be considered clinically significant by the patient was a reduction of 5 points or more in the VAS score and 27 points or more in the ODI score respectively. Thirty-two patients (74.4%) showed a clinically significant reduction of pain and twenty-seven (62.8%) reported a clinically significant improvement of disability. Post-operative complications were temporary (less than 24 h) and included 1 case of minor bleeding (2.3%), 1 case of vesical retention (2.3%), 6 cases of an epidural block (14%), and 5 cases of steroid side effects (11.6%).

Table 2 shows the variables associated with pain recurrence after FJI for LBP patients. There was a trend for pain recurrence in patients with sedentary lifestyles (HR 2.43, CI95% 0.88–6.24, *p* = 0.09). Increased levels of presurgical anxiety (BAI scores > 3) showed and greater association with pain recurrence than patients with lower anxiety levels (HR 2.71, CI95% 1.01–7.25, *p* = 0.05). Presurgical catastrophizing (PCS score ≥ 5, adjusted HR 4.4, CI 95% 1.7–11.3, *p* = 0.002) and smoking (Adjusted HR 12.5, CI 95% 1.1–138.9, *p* = 0.04) were predictors of pain recurrence. There was only 1 smoking patient (more than 40 cigarettes per day for 20 years) who showed any improvement with the procedure.

**TABLE 2:** Univariate cox regressions showing the variables associated with pain recurrence in 43 patients with LBP treated with FJI.

**Variables**	**HR**	**95% CI**	***p***
Age ≥ 50 years	1.0	(0.4–2.3)	0.93
Male	1.1	(0.4–2.3)	0.89
BMI > 30 (kg m^–2^)^a^	1.1	(0.4–3.2)	0.83
Scholarship < 12 years	1.1	(0.5–2.5)	0.80
Manual work	1.2	(0.5–3.1)	0.62
Smoking	20.6	(1.8–227.8)	0.01
Diabetes Mellitus II	2.0	(0.3–14.9)	0.51
Fibromyalgia	1.9	(0.7–5.3)	0.17
Hypertension	1.0	(0.4–2.3)	0.95
Sedentary lifestyle	2.3	(0.8–6.2)	0.09
Previous opioid use	1.1	(0.4–2.6)	0.78
Previous lumbar surgery	1.0	(0.4–2.8)	0.89
Time of pain duration ≥ 24 m	1.1	(0.5–2.5)	0.75
**MRI findings^b^**			
Facet arthropathy ≥ 3 levels	1.2	(0.3–1.8)	0.60
Spine deformity	1.1	(0.3–1.6)	0.60
DDD^c^	1.7	(0.5–5.9)	0.35
Disc herniation	1.0	(0.4–2.5)	0.88
Spondylolisthesis	1.3	(0.5–3.4)	0.50
Facet synovial cyst	1.1	(0.1–8.6)	0.88
Lumbar muscle weakness	1.4	(0.6–3.6)	0.42
Supraspinal ligament stretch	1.0	(0.4–2.4)	0.94
Spinal stenosis	1.1	(0.4–2.6)	0.79
**Psychiatric Symptoms**			
Catastrophizing, PCS^d^ ≥ 5	4.6	(1.8–11.7)	0.001
Anxiety, BAI^e^ ≥ 10	3.0	(1.4–6.7)	0.007
Depression, BDI^f^ ≥ 10	3.9	(1.6–9.6)	0.003

*HR = Hazard ratio; CI = Confidence interval; ^*a*^Body mass index; ^*b*^MRI = Magnetic resonance imaging; ^*c*^Degenerative disc disease; ^*d*^Pain Catastrophizing Scale; ^*e*^Beck Anxiety Index; ^*f*^Beck Depression Index.*

Table 3 shows the final results of multiple Cox regression analyses demonstrating that catastrophizing (PCS score ≥ 5) and smoking, but not sedentary lifestyle and anxiety symptoms remained independently associated with pain recurrence after FJI.

**TABLE 3:** Multivariate cox regression model showing the independent predictive variables of pain recurrence in 43 patients with LBP treated with FJI.

**Predictive variables**	**Adjusted HR for**	**CI 95%**	**“*p*” level**
	**pain recurrence**		
**Initial model**			
Sedentary lifestyle	1.6	(0.6–4.6)	0.35
Smoking	6.6	(0.5–86.7)	0.15
Fibromyalgia	1.3	(0.4–4.4)	0.65
Catastrophizing symptoms, PCS ≥ 5	4.0	(1.3–10.2)	0.02
Anxiety symptoms, BAI ≥ 10	0.8	(0.4–3.8)	0.77
Depression symptoms, BAD ≥ 10	1.4	(0.4–5.5)	0.61
**Final model**			
Smoking	12.5	(1.1–138.9)	0.04
Catastrophizing symptoms, PCS ≥ 5	4.4	(1.7–11.3)	0.002

*Adjusted HR = Adjusted hazard ratio; CI 95% = confidence interval; PCS = Pain Catastrophizing Scale; BAI = Beck Anxiety Index.*

**FIGURE 2:**
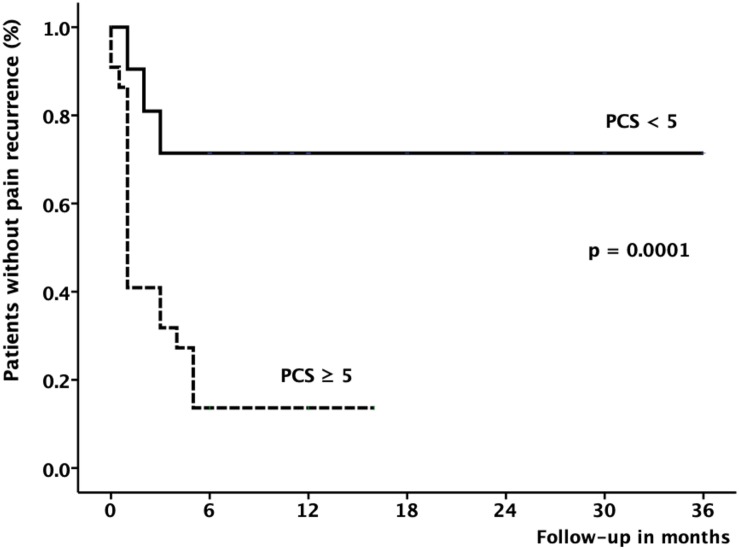
Kaplan–Meier survival curve shows the results of analysis of pain recurrence rate (*Y*-axis) for patients with LBP treated with FJI during 6-month of follow-up (*X*-axis). The curve indicates that 71.4% of the patients with PCS < 5 remains pain-free after the FJI in comparison to 13.6% of patients showing elevated levels of catastrophizing symptoms (PCS ≥ 5), *p* < 0.0001 by log-rank test.

Figure 2 shows the Kaplan–Meier survival curve demonstrating the cumulative percentage of patients remaining without pain after the FJI for LBP according to the PCS score. Pain recurrence occurred in 25 patients (58.1%). However, only 29% percent of patients with lower levels of catastrophizing (PCS < 5, continuous line) showed pain recurrence compared with 86.4% of recurrence among those with increased levels (PCS ≥ 5, dashed lines) of catastrophizing (Cox regression, “*p*” = 0.0001). In all cases of failure, the recurrence of pain occurred up to 6 months after the procedure.

## Discussion

This study examined pain relief efficacy and the independent predictors for pain recurrence in 43 patients with chronic non-specific LBP who underwent FJI. The results show that FJI significantly reduces pain intensity (VAS scale) and that 81.4% of patients reported good to excellent results 6 months after the procedure based on MacNab criteria. Also, within the same follow-up period, there was a significant improvement in pain perception (VAS) and disability (ODI). Pain recurrence was more associated with patients showing higher catastrophizing scores pain recurrence after the FJI.

Although chronic pain typically starts as a biological mechanism, psychological and social factors often play critical roles in the development and maintenance of chronic pain (Macnab, 1971; Sarda et al., 2012). Emotional symptoms modulate and amplify the pain experience, affecting the success of the treatment for chronic pain (Gatchel et al., 2007). Previous studies have observed that corticospinal excitability is modulated by anxiety favoring the loss of descendent inhibitory influx (Jeans and Toman, 1956; North et al., 1996; Vidor et al., 2014). As showed by some authors, neither anxiety (BAI) nor depression (BDI) symptoms showed a correlation with pain recurrence after FJI for chronic non-specific LBP.

However, in our patients, the level of pain catastrophizing was the only predictor of pain recurrence after FJI. Even the applied cut-off of PCS ≥ 5 is considered very low for this psychological measurement, studies have shown that catastrophizing is the most important psychological factor associated with the onset and maintenance of chronic pain. There is a substantial amount of evidence suggesting that pain catastrophizing may contribute to a negative emotional status as well as hypervigilance, which is an essential factor for a negative response to the pain treatment (North et al., 1996; Sullivan et al., 2001; Jensen et al., 2007; Pulvers and Hood, 2013; Rosenberg et al., 2015; Terry et al., 2015). Characteristically, pain catastrophizing can increase pain perception due to a negative amplification of pain-related thoughts through rumination (repetitive thoughts about pain), magnification (exaggerated concern about negative consequences of pain), and helplessness (believing nothing will change the pain) (Sullivan et al., 2001; Pulvers and Hood, 2013; Campbell et al., 2015). In other words, catastrophizing may increase pain intensity as patients maintain attention to their pain sensation, which results in rumination and subsequent magnification of painful sensations.

Catastrophizing is also likely to interrelate with the decreased perception of control over pain and ability to decrease pain. An increased catastrophizing score during experimental pain is associated with lower activation of descending pain-inhibitory controls (Goodin et al., 2009). Moreover, studies have shown that catastrophizing is associated with increased brain activity in regions associated with anticipation of pain (medial frontal cortex, cerebellum), attention to pain (dorsal anterior cingulate gyrus, rostral anterior cingulate cortex, dorsolateral prefrontal cortex), emotional aspects of pain (claustrum, closely connected to amygdala), and motor activity (Gracely et al., 2004; Seminowicz and Davis, 2006). These studies of neural pathways have fueled tremendous excitement in the efforts to elucidate the mechanism underlying pain perception mediated by catastrophizing.

Although it is well-known that chronic LBP is ubiquitous among obese patients (Atchison and Vincent, 2012; Samartzis et al., 2012), in the present study, there was no association between BMI and FJI outcome. However, only three cases (7%) had BMI > 30, and all with less than 31. Therefore, the small number of obese patients does not exclude a possible type II error in our study regarding the non-association observed between BMI and MCI. Besides, obesity may affect pain aspects not only due to mechanic aspects but also due to its relationship with other aspects of disability. Therefore, we believe the role of BMI as a variable to improve prognostic models for pain recurrence after FJI require further studies.

As previously described by other authors (Manchikanti et al., 2008), there was no significant association between pain recurrence and pharmacological treatment with opioids before undergoing FJI. With regards to previous lumbar surgery, although studies (Manchikanti et al., 2007; DePalma et al., 2012) have presented a lower prevalence of facet joint pain in patients who underwent spinal surgical interventions, this factor showed no significant difference in our study. Regarding the influence of smoking, although only a female patient was a smoker, she described no improvement earlier after the FJI, which was in contrast with all other patients, who reported pain recurrence occurring at least 1 month after the procedure. Even considering that this finding remains to be confirmed by further studies with a higher number of more smokers, the negative impact of smoking on the LBP response to IJF have biological plausibility. The mechanisms may involve vasoconstriction reducing the perfusion and nutrition of the intervertebral discs, interfering with healing. Smoking is associated with osteoporosis, and increases the level of circulating pro-inflammatory cytokines leading to amplification of pain (Shiri et al., 2010). Finally, smoking changes down-regulates collagen genes and up-regulates aggrecan and the tissue inhibitor of metalloproteinase-1 genes in the intervertebral disks (Manchikanti et al., 2007). There is no study showing the association between smoking and LBP response and recurrence after FJI. However, in standard open discectomy for subacute/chronic symptomatic lumbar disc herniation, smoking is independently associated with pain recurrence (Suri et al., 2017).

This study has some limitations. Psychometric scales (BAI, BDI, PCS) were only applied pre-operatively. However, psychological symptoms after FJI also may affect the perception of pain and disability and should be considered for evaluation in future studies. The small sample size indeed reduced the power of our analysis, and the reader should consider the possibility of false-negative results (type II errors) in our multiple Cox analysis. Further studies, including a higher number of smokers, would be desirable to clarify the relevance of this variable for LBP recurrence after FJI. However, a significant association found in a small sample supports the positive results’ credibility. The lack of examiner blinding for the evaluated endpoint is also a limitation. Our study design does not permit to exclude the placebo effect involved in the reduction of subjective outcomes such as pain, which can be even higher for procedures than for medications (Kong et al., 2007). Although the ODI analyzes aspects of the ability to manage in everyday life, the scores are dependent on the patients’ report. The inclusion of a control group in a randomized controlled trial design helps to control these limitations. In the last year, after we finished our patient inclusion, a randomized placebo-controlled trial showed no differences in pain reduction, medication reduction and ODI improvement at 1 month after intra-articular facet injections with bupivacaine and steroid, MBBs, or saline. Interestingly, in the same study, patients who underwent facet injections and had a positive block in the immediate post-procedural period also had a better outcome after the subsequent radiofrequency ablation (Cohen et al., 2018). Because all patients included in our study received the same treatment, we hypothesized that the catastrophizing symptoms could indicate, at least in part, differences in the susceptibility to the placebo effect among our patients. This hypothesis could only be tested analyzing the association between catastrophizing symptoms and the pain outcome in the placebo group recruited in a randomized placebo-controlled trial.

Conversely, positive aspects of our study were: (i) the prospective study design; (ii) the extensive control applied to imbalances in the demographic, clinical, surgical and radiological variables including the psychological and emotional profiles of the patients; (iii) the multivariate analysis approach, using the Cox regression analysis; and (iv) a minimum of a 6-month follow-up period.

To summarize, the level of pain catastrophizing is a relevant predictor of pain recurrence after lumbar FJIs for LBP. Based on these findings, the management of catastrophizing before FJI for LBP could contribute to reducing pain recurrence and thus deserves clinical attention. Identify these dysfunctional beliefs before therapeutic intervention would require a psychoeducational intervention aiming to reduce these beliefs and contributing to a better outcome. This study also underscores the importance of a multidimensional approach for LBP treatment. Developing an improved approach for catastrophizing management in LBP patients is essential and should be investigated in further controlled trials. If confirmed in other populations, the evaluation of catastrophizing may become a useful tool for physicians and patients to use in their decision making about surgical treatment of LBP.

## Data Availability

All datasets generated for this study are included in the manuscript and/or the supplementary files.

## Ethics Statement

RB approval/Research Ethics Committee: Research approved by the Ethics Committee of our Institutions (*Plataforma Brasil CAAE 34317214.1.3001.5360/CEP-UFSC 832.267*), and all patients gave written informed consent.

## Author Contributions

WC designed the procedures and involved in data collection, pre- and post-operative questionaries, statistical analysis, and manuscript edition. ML reviewed the English version of the manuscript and collected the data. JS did psychological analysis. AS involved in logical analysis and reviewed the manuscript. KL edited the manuscript. AL wrote the “Materials and Methods” section. RW involved in statistical analysis and contributed for review and conclusions.

## Conflict of Interest Statement

The authors declare that the research was conducted in the absence of any commercial or financial relationships that could be construed as a potential conflict of interest.
